# Single‐cell transcriptomic profiling of maize cell heterogeneity and systemic immune responses against *Puccinia polysora* Underw

**DOI:** 10.1111/pbi.14519

**Published:** 2024-11-29

**Authors:** Xiao‐Cui Yan, Qing Liu, Qian Yang, Kai‐Lai Wang, Xiu‐Zhen Zhai, Meng‐Yun Kou, Jia‐Long Liu, Shang‐Tong Li, Shu‐Han Deng, Miao‐Miao Li, Hui‐Jun Duan

**Affiliations:** ^1^ State Key Laboratory of North China Crop Improvement and Regulation Key Laboratory of Crop Germplasm Resources in North China, Ministry of Education, College of Agronomy Hebei Agricultural University Baoding Hebei China; ^2^ State Key Laboratory of North China Crop Improvement and Regulation Key Laboratory of Vegetable Germplasm Innovation and Utilization of Hebei, College of Life Sciences Hebei Agricultural University Baoding China; ^3^ Glbizzia Biosciences Beijing China; ^4^ Key Laboratory of Seed Innovation, Institute of Genetics and Developmental Biology Chinese Academy of Sciences Beijing China

**Keywords:** Single‐cell Transcriptomic, *Puccinia polysora* Underw, Resistance differences, *ZmChit7*, Molecular marker, Maize

## Abstract

Southern corn rust (SCR), caused by *Puccinia polysora* Underw (*P. polysora*), is a catastrophic disease affecting maize, leading to significant global yield losses. The disease manifests primarily as pustules on the upper surface of corn leaves, obscuring our understanding of its cellular heterogeneity, the maize's response to its infection and the underlying gene expression regulatory mechanisms. In this study, we dissected the heterogeneity of maize's response to *P. polysora* infection using single‐cell RNA sequencing. We delineated cell‐type‐specific gene expression alterations in six leaf cell types, creating the inaugural single‐cell atlas of a maize leaf under fungal assault. Crucially, by reconstructing cellular trajectories in susceptible line N110 and resistant line R99 during infection, we identified diverse regulatory programs that fortify R99's resistance across different leaf cell types. This research uncovers an immune‐like state in R99 leaves, characterized by the expression of various fungi‐induced genes in the absence of fungal infection, particularly in guard and epidermal cells. Our findings also highlight the role of the fungi‐induced glycoside hydrolase family 18 chitinase 7 protein (ZmChit7) in conferring resistance to *P. polysora*. Collectively, our results shed light on the mechanisms of maize resistance to fungal pathogens through comparative single‐cell transcriptomics, offering a valuable resource for pinpointing novel genes that bolster resistance to *P. polysora*.

## Introduction

Maize (*Zea mays* L.) is the most important staple food and feed crop, playing an indispensable role in world food security. However, its production is currently threatened by various diseases and pests, notably Southern Corn Rust (SCR), caused by *Puccinia polysora* Underw (*P. polysora*). This devastating foliar disease has the potential to inflict yield losses ranging from 50% to complete crop failure during severe outbreaks (Brewbaker *et al*., 2011; Liu *et al*., 2016). Such severe yield losses have been reported across >110 countries spanning Africa, Asia, the Americas and Australasia (CABI, 2021; Sun *et al*., 2021). Discovery and utilization of SCR resistance genes in maize breeding programs is the most economical and preferred approach to fend off the pathogen. Currently, there is insufficient knowledge underlying the molecular mechanisms and key genes of resistance to corn rust disease. Additionally, our understanding of the alternate host of *P. polysora* and the roles of teliospores in host‐pathogen interactions is inadequate. The undefined resistance pathway and long‐term traditional breeding methods cause great difficulties in the development of superior cultivars (Debnath *et al*., 2019; Sun *et al*., 2021). Therefore, there is an urgent need to elucidate the molecular regulatory network governing maize‐pathogen interactions, identify defence or resistance‐associated genes and proteins, and innovate molecular breeding techniques to enhance disease resistance.

High‐resolution single‐cell RNA sequencing (scRNA‐seq) analysis offers cell‐type specific molecular insights, shedding light on the biological functions of genes and the intricate molecular mechanisms governing their expression (Kulkarni *et al*., 2019; Potter, 2018; Rich‐Griffin *et al*., 2020). With this technology, it is possible to explore the heterogeneity among different cell types in complex tissues and to identify unknown cell types. In maize, single‐cell transcriptomics has predominantly been applied to study developmental processes in the maize ear, differentiation of bundle sheath (BS) cells, shoot apical meristem (SAM) stem cells and the cis‐regulatory landscape (Bezrutczyk *et al*., 2021; Marand *et al*., 2021; Satterlee *et al*., 2020; Xu *et al*., 2021). However, there are scant reports on the interaction between corn and pathogens using single‐cell transcriptomics analysis, despite fact that the plant responses to pathogens were dynamic and heterogeneous among cell types.

In this study, we utilized scRNA‐seq technology to construct the first transcriptomic atlas of maize leaf tissue infected with *P. polysora*. This enabled the identification of pathogen‐responsive cell clusters, delineating immune, transitional and susceptible states. A pseudotime trajectory analysis has mapped the disease progression continuum, from robust immunity to heightened susceptibility. We have pinpointed cluster‐specific marker genes for each maize leaf cell type and through analysing expression patterns, have decoded pathways and genes integral to disease resistance. Notably, the abundance of one Xylanase inhibitor protein 1‐like, ZmCHit7, was significantly greater in the infected maize leaves. The functional markers identified in the infected atlas hold promise for marker‐assisted selection in maize breeding, potentially revolutionizing disease resistance strategies.

## Results

### The single‐cell leaf transcriptomic landscape of R99 and N110 under *P. polysora* infection

**FIGURE 1:**
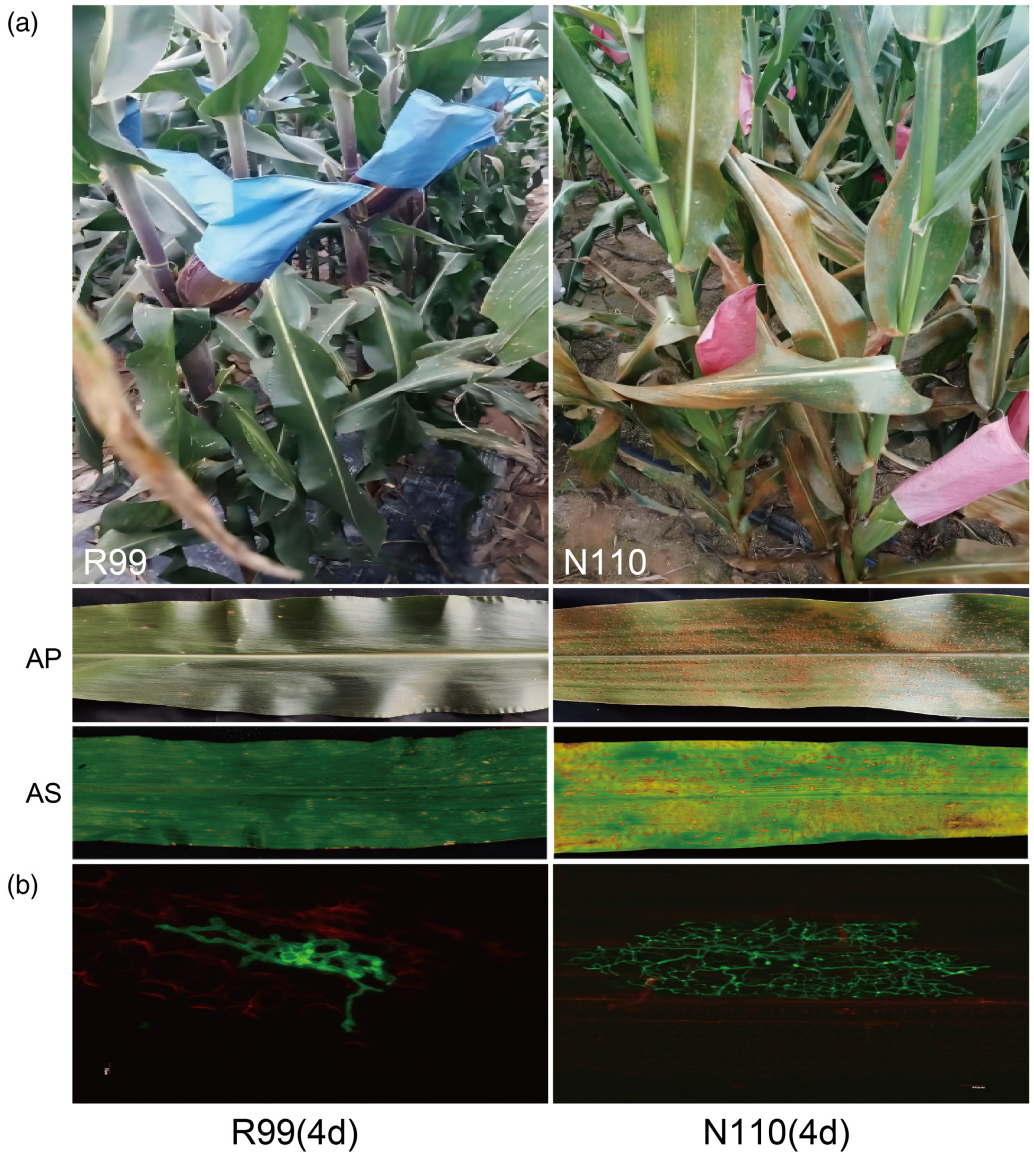
The single‐cell leaf transcriptomic landscape of R99 and N110 under *P. polysora* infection. (a) Field trials and seedling tests reveal the contrasting responses of R99 (resistant) and N110 (susceptible) to *P. polysora* infection. AP: Adult‐plant stage phenotypic responses; AS: Seedling stage reactions. (b) The lower panel displays fungal structures at 4 days post‐inoculation (dpi).

To understand the interaction between maize and fungal diseases, we first compared the phenotypic differences between maize cultivars R99 and N110 before and after inoculation with *P. polysora*. The results indicate significant differences in phenotypes between two cultivars following *P. polysora* infection. Specifically, R99 was resistant to infection types IT 1, whereas N110 was susceptible to IT 7–9 (Figure 1a). Moreover, there was significantly reduced mycelial growth in R99 seedlings at 4 days post inoculation (dpi), consistent with later observations based on infection type (Figure 1b). Therefore, R99 exhibits a resistance phenotype, while N110 is more sensitive at all stages under *P. polysora* infection (Figure 1).

**FIGURE 2:**
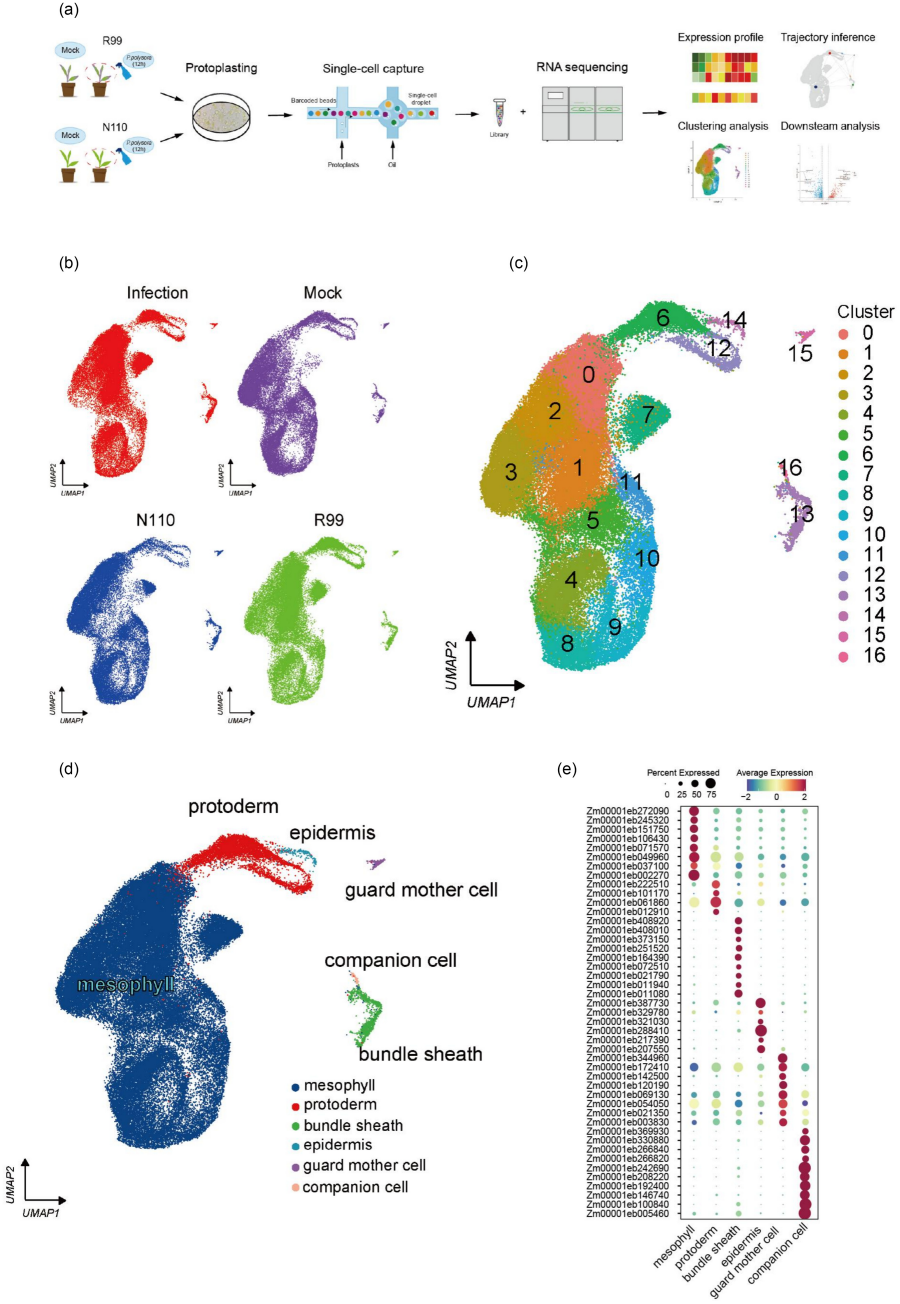
Cell types identified in maize leaves based on scRNA‐seq. (a) Overview of the scRNA‐seq experiment. Protoplasts were isolated from 1 to 5 mm leaves of maize plants (R99 and N110) at 12 h after the *P.polysora* and mock inoculations, respectively. scRNA‐seq libraries were generated using the DNBelab C4 platform followed by high‐throughput sequencing. Analysis workflow including removal of low‐quality cells, gene expression normalization, calculation of variable genes, clustering and functional verification of candidate genes. (b) UMAP visualization of scRNA databases of maize leaves at the infected and Mock condition. (c) UMAP visualization of the 17 cell clusters identified by unsupervised clustering analysis. (d) Built on cells from the clusters 6 and 17 with the colours representing sub‐clustering. Each dot represents a single cell. Colours of dots are corresponding to cell clusters. The cell atlas with cell type annotation with the colours representing the 6 major cell types based on expression of cell type marker genes. (e) Proportions and average expression levels (scaled) of selected marker genes for six cell types.

To elucidate the molecular and cellular mechanisms underlying phenotypic differences between two cultivars under *P. polysora* infection, we constructed the single‐cell atlas of R99 and N110 leaves under mock and fungal infection conditions, sampled at 12 h post‐inoculation (Figure 2a). Firstly, we isolated protoplasts from inoculated or mock‐treated maize third leaf tissues (R99 and N110), in two biological replicates (Figure S1a; Table S1). After filtering out low‐quality cells, we obtained a total of 70 803 high‐quality single cells from all samples (Table S2). Due to highly similar gene expression patterns across replicate experiments (r = 0.79–0.99, Figure S1b,c), the two biological replicates will be merged for downstream analysis (Figure 2b). Subsequently, we performed standardization, normalization, dimensionality reduction and clustering on all cells, and identified 17 major cell clusters (Figure 2c).

Furthermore, based on well‐known cell type specific markers from published articles related to corn in leaf (Abe *et al*., 2003; Baker *et al*., 2016; Bezrutczyk *et al*., 2021; Cao *et al*., 2014; Chang *et al*., 2012; Fu *et al*., 2018; Gao *et al*., 2017; Li *et al*., 2018, 2022; Liu *et al*., 2020, 2021a; Marand *et al*., 2021; Okumoto *et al*., 2002; Ortiz‐Ramirez *et al*., 2021; Xiao *et al*., 2018; Xu *et al*., 2021), we identified biologically significant cell types by cluster‐specific marker genes (Figure 2d,e; Table S3). These 17 clusters are classified into six major cell types: mesophyll cells (cluster 1–5 and cluster 7–10), protoderm (cluster 6 and 12), bundle sheath (cluster 13), epidermis (cluster 14), guard mother cell (cluster 15); and companion cell (cluster 16) (Figure 2c,d). Among them, mesophyll cells comprised the majority, accounting for 82.22% to 95.79%, followed by protoderm, accounting for 1.95% to 15.94%. The remaining cell types, including epidermis, bundle sheath, guard mother cell and companion cell presented a small proportion, ranging from 0.03% to 2.57% (Figure S2; Table S2). There is, in addition, one further point to make. Novel cell type‐specific makers were also shown in the heatmap of the top 10 enriched genes in each cell types (Figure S3; Table S4). Collectively, the maize leaf cell atlas contributes to the further characterization of the fundamentals of these specific cell types and captured dynamic gene expression changes under *P. polysora* infection across R99 and N110.

### Cell type specific transcriptomic changes under *P. polysora* infection across R99 and N110

**FIGURE 3:**
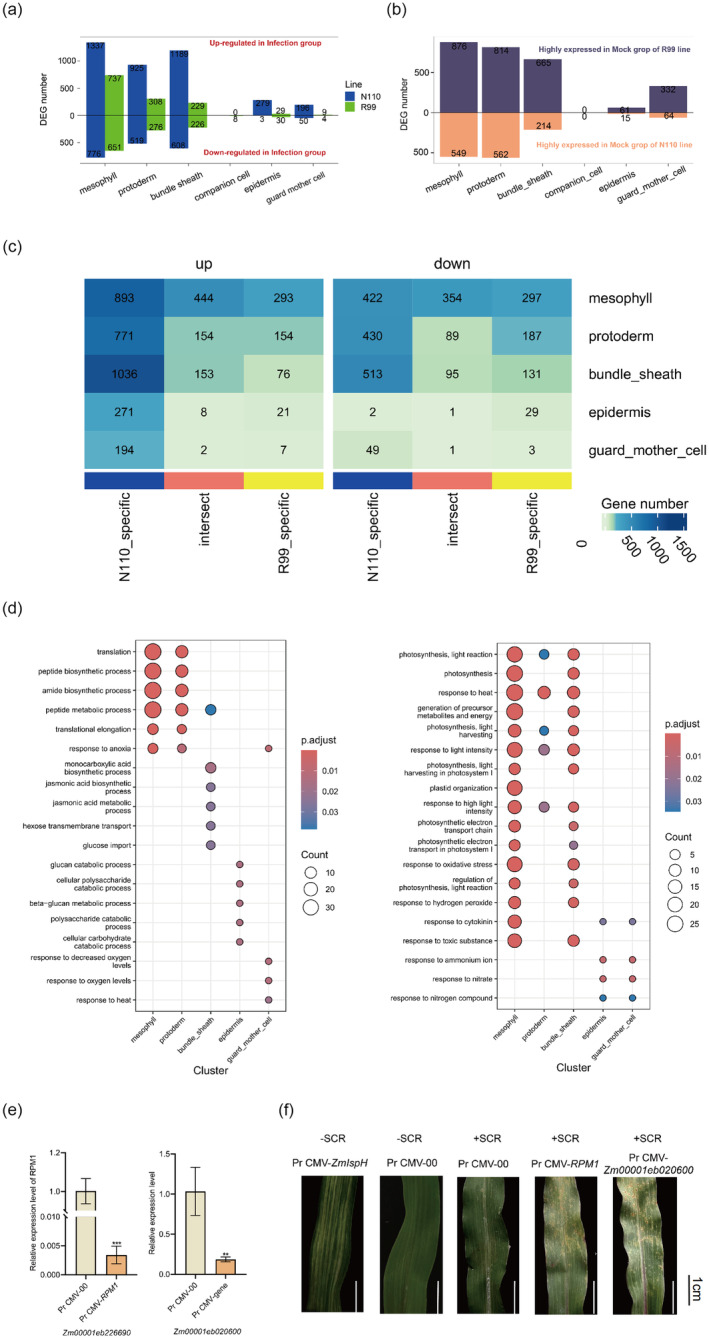
Cell type‐specific differentially expressed genes (DEGs) under *P. polysora* infection across R99 and N110. (a) The bar plot shows the number of up and down DEGs at infected group for each cell type of N110 and R99, compare to the mock condition. (b) The bar plot shows the number of up and down DEGs of R99 at mock condition, compare to the N110. (c) Heatmap shows the number of shared and cultivar‐specific expressed genes for each cell‐type cluster between R99 and N110. The numbers on the graph represent the number of DEGs. (d) Scatter plots shows GO enrichment analysis of shared genes across all cell types between N110 and R99. (e) RT‐PCR results showing the efficient VIGS of *Zm00001eb020600* and *Zm00001eb0226690*. (f) Representative images of *P. polysora* spores in the in control and VIGS (*Zm00001eb020600* and *Zm00001eb0226690*) maize leaves upon infection.

Different cell types react differentially to pathogen infection, which could be captured by analysing cell type specific differentially expressed genes (DEGs). When infected by *P. polysora*, the number of differentially expressed genes varies across cell types. This heterogeneity may reflect different response patterns of cell types to pathogen infection. Interestingly, we found the number of the DEGs under *P. polysora* are dramatically reduced in the resistant line R99 compared to N110, suggesting systematic attenuation of the influence on the R99 transcriptome under infection (Figure 3a). N110 (Infection vs. Mock) was found to have a total of 5882 DEGs, including 3926 upregulated genes and 1956 downregulated (Figure 3a). Additionally, we found 45 upregulated and 2 downregulated genes (*Zm00001eb021590* and *Zm00001eb383680*) were identified in all but the companion cell (Figure S4a). In contrast, 2057 DEGs were identified in R99 (Infection vs. Mock), with 1312 upregulated and 1195 downregulated genes (Figure 3a). Furthermore, we also found that 4 upregulated and 1 (*Zm00001eb383680*) downregulated genes were identified in all but the companion cell samples (Figure S4b). These results suggest cell type specific changes under *P. polysora* infection and the smaller number of DEGs upon *P. polysora* infection in R99 indicates a significant attenuation of the transcriptomic response in the resistant line R99, which may be due to R99 being in an immune state and less affected by external factors.

To further explore such speculation, we compared the transcriptomic differences between R99 and N110 in mock condition. Overall, the R99 displays a total of 2748 upregulated and 1404 downregulated genes compared to N110 and the results are shown in Figure 3b,c (Table S5). We further focus on and surveyed key resistance‐related genes in the co‐regulated genes in both N110 and R99. And found that 1301 DEGs were shared among the two maize varieties (N110 and R99), 761 of which were co‐upregulated and 540 genes were co‐downregulated in the certain cell type(s) during fungal infection (Figure 3c). Meanwhile, we also subjected all co‐expressed DEGs to GO and KEGG pathway enrichment analysis to characterize the metabolic pathways in each cell type(s) of lines R99 and N110 inoculated with *P. polysora* (Figure 3d). For example, upregulated‐genes with confirmed or predicted roles in ribonucleoprotein (GO:0006412), peptide biosynthetic process (GO:0043043) and defence response to fungus (GO:0050832) were enriched in cells of the mesophyll and protoderm; upregulated‐genes related to inorganic ion transmembrane transport (GO:0098660), jasmonic acid biosynthetic process (GO:0009695) and metabolic process (GO:0008152) were enriched in the bundle sheath; And related to cellular polysaccharide catabolic process (GO:0000272), carbohydrate catabolic process (GO:0016052) and carbohydrate metabolic process (GO:0005975) were enriched in the epidermis. However, down regulated genes related to response to heat (GO:0009408) and photosynthesis (GO:0015979) were enriched in cells of the mesophyll, protoderm and bundle sheath and related to response to ammonium ions (GO:0060359), nitrate (GO:0010167) and cytokinin (GO:0009735) were enriched epidermis and guard cell. The results showed that the responsive mechanisms of R99 and N110 to *P. polysora* demonstrated obvious difference in cell clusters.

We have identified 14 and 15 *NLR* genes which are induced in R99 and N110 upon SCR infection, respectively (Figure S5a). Among them, *Zm00001eb020600* was highly expressed in R99 compared to N110 in guard cells, consistent with our working model that R99 was in a pre‐immune state. *Zm00001eb0226690* (*RPM1*) were induced by SCR infection in mesophyll cells in both R99 and N110 (Figure S5b–d). We performed virus induced gene silencing (VIGS) on these *NLR* genes upon SCR infection (Figure 3e) and found more fungal spores when these two *NLR* genes were silenced (Figure 3f), suggesting that these two *NLR* genes positively contribute to the resistance to SCR.

### Guard cells of R99 were resistant to *P. polysora* infection

**FIGURE 4:**
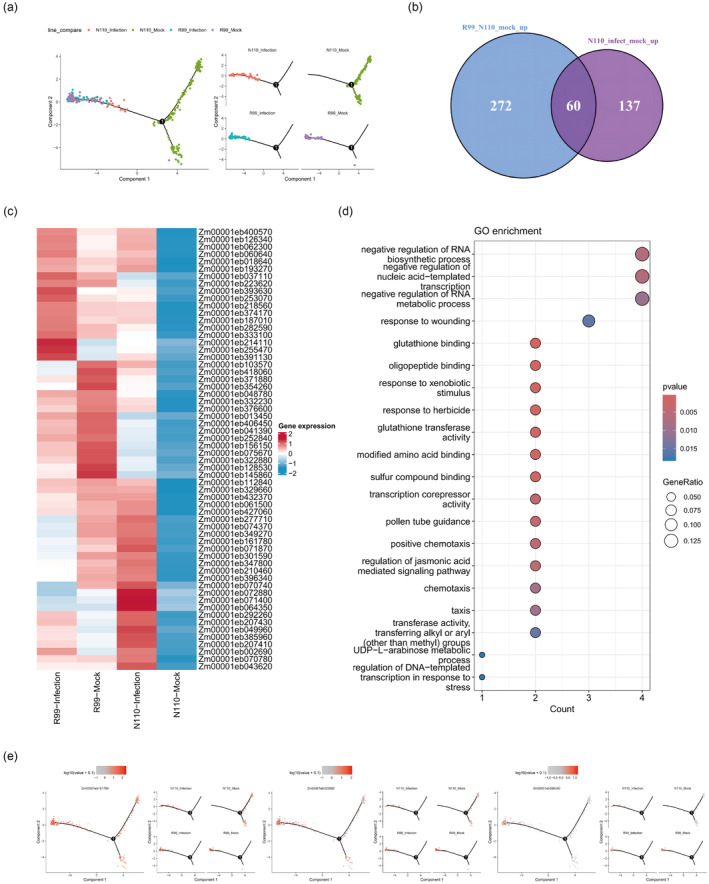
Guard cells of R99 were resistant to *P. polysora* infection. (a) Pseudotime trajectory of guard cells inferred by Monocle 2 from left to right, representing all cells and different groups. Each dot indicates a single cell. (b) Venn diagram shows the intersections of up – regulated DGEs by Mock (R99 vs. N110) and N110 (infection vs. mock). The blue cycle represents genes highly expression in R99 compared toN110 in the mock condition, the purple cycle represents genes up‐regulated in N110 upon infection. (c) Heatmap shows the expression of 60 intersected genes mentioned in (b) across the four conditions. Each row represents one gene. (d) GO enrichment analysis of 60 intersected genes in (b). (e) Expression patterns of cell‐type marker genes mapped on the pseudotime trajectory of guard cells, with colour intensity indicating expression levels in individual cells.

To demonstrate the immune‐like state at higher resolution, we reconstructed the gene expression trajectory upon infection for cell type by pseudotime analysis using both infected and mocked R99 and N110 cells. In the guard cells, across the pseudotime trajectory, we observed a gradual shift of mock to infected cells from the right end to the left end in the N110 line, suggesting the responses processing of guard cells to *P. polysora* infection (Figure 4a). Surprisingly, guard cells of the R99 line, even in the mock condition, clustered at the trajectory's left end, suggesting an inherent immune‐like state that may contribute to the resistance to *P. polysora* in R99. The results have proved again that R99 activates the immune state prematurely in the mock state and the DEGs across the state transition from mock to infected cells suggest genes/pathways *P. polysora* are induced by fungi infection.

To further clarify the immune‐like state of R99, we compared the gene expression differences between R99 and N110 in a mock sample. Among the 197 genes that are induced by infection in N110, we found a total of 60 genes are highly expressed in R99 compared to N110 in mock condition (Figure 4b,c). A further examination of the 60 genes revealed GO terms related to multiple stress response pathways, including plant hormone signalling pathways and genes associated with disease resistance (Figure 4d). Such as, genes encoding ATP‐binding cassette transporters (ABC transporters), Ca^2+^‐calmodulin (CaM), CEBIP, GST and the transcription factors WRKY, ZIM and MYB play a crucial role in plant innate immune responses (PTI and ETI) (Dodd *et al*., 2010; Van Wersch *et al*., 2020). ATP‐binding cassette (ABC) transporters play an important role in pathogen resistance (Sun *et al*., 2022). This prompted us to investigate the correlation between the gene expression levels of ABC‐related genes in the guard cells and their pseudotime values. Key genes, which has been reported to participate in pathogen resistance in other crops, such as *PDR* (*Zm00001eb396340*), *ABCG36* (*Zm00001eb161780*) and *ABCG43* (*Zm00001eb322880*) (Aryal *et al*., 2023; Zhang *et al*., 2020), exhibited a progressive increase in expression in guard cells with pseudotime values trending toward left, signifying a gradual induction in cells at the infection sites (Figure 4e).

Besides, previous research has proved that *NBS‐LRR* genes play an important role in disease resistance of the plant (Kedzierski *et al*., 2004; Lv *et al*., 2023). Therefore, based on the maize *NBS‐LRR* gene database (Liu *et al*., 2021b), a total of 204 *NBS‐LRR* genes were detected to be expressed in our expression profiles (Figure S6; Table S6). Among them, 7 upregulated DEGs were annotated as *NLR* and 3 downregulated DEGs in the mock group (R99 vs. N110) (Table S7). In the present study, known as *NBS‐LRR* genes, the transcript abundance of *RPM1* (*Zm00001eb226690*) was significantly upregulated only in the guard cells of R99 (Figures S5b–d and S6). These findings suggest that R99 guard cells inherently express a suite of immune‐responsive genes, maintaining elevated levels of PTI and ETI, even in the absence of fungi infection, thereby bolstering resistance against pathogen *P. polysora*.

### Epidermis cells of R99 contributes to resistance to *P. polysora* infection

**FIGURE 5:**
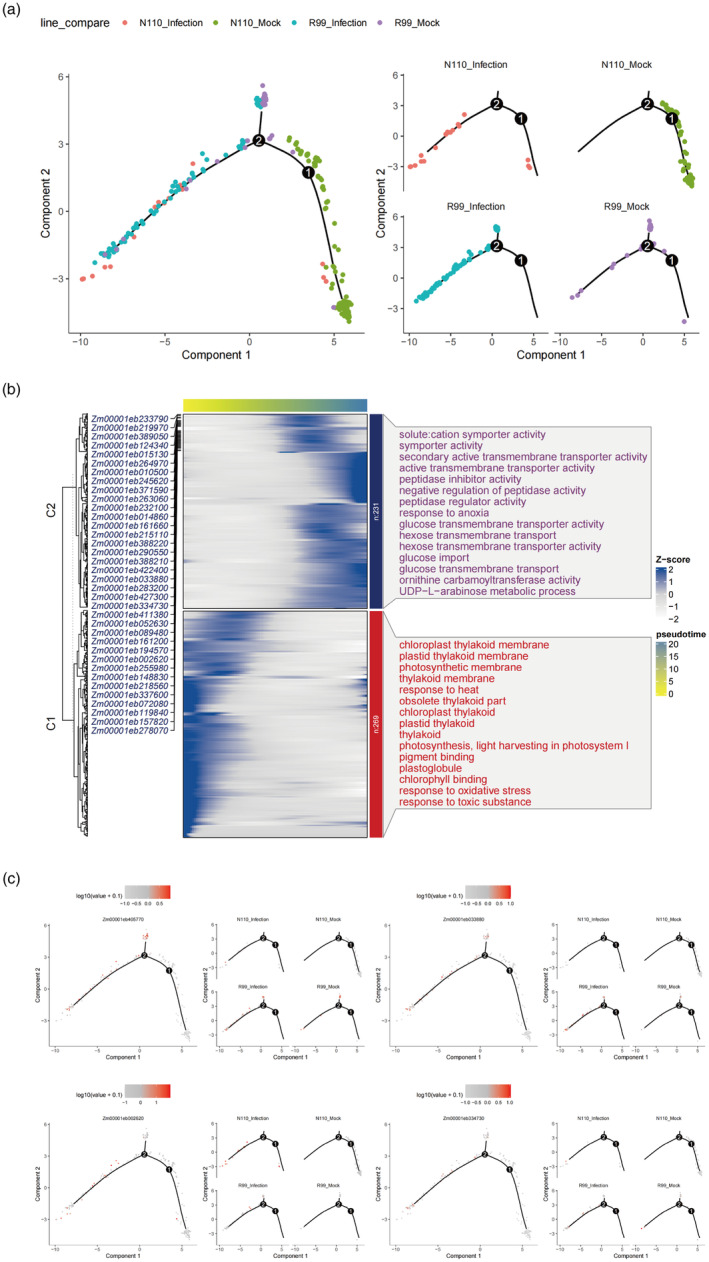
Epidermal cells of R99 were resistant to *P. polysora* infection. (a) Pseudotime trajectory of epidermis cells inferred by Monocle 2 From left to right, representing all cells and different groups. Each dot indicates a single cell. (b) Smoothed expression heatmap of the top500 altered genes of epidermis cell along the differentiation trajectory from Mock to infection condition. The x‐axis represents cells ordered by pseudotime (from left to right) and different colours correspond to the scaled (Z‐scored) expression of each gene in each cell. the altered genes were clustered into two clusters with distinct expression patterns. C1 genes are enriched in cells on the primate of the trajectory and C2 genes are enriched in cells on the end of the trajectory. GO terms for each cluster are shown on the right. (c) Expression of the representative of different genes on the pseudotime trajectory.

Epidermal cells act as the first layer of defence against plant diseases. To assess the expression profiles of maize leaves at the early and late stages of *P. polysora* infection, we also performed a pseudotime analysis of epidermal cells after *P. polysora* infection like the case in guard cells. We found that the trajectory of N110 epidermis cells shifted gradually from right to left during the responsive process after infection. Like that in guard cells, the epidermal of R99 highly expressed genes induced by *P. polysora* infection in mock conditions, suggesting a pre‐activated defence state (Figure 5a). Cluster analysis of genes with significant expression changes across pseudotime (Top500 genes) identified two distinct patterns: one associated with the normal physiological functions of epidermal cells, such as photosynthesis and light response (Cluster 1) and another associated with stress response and nutrient utilization, indicative of a defence response (Cluster 2) (Figure 5b). In cluster 1, genes are highly expressed in N110 mock samples and in cluster 2, genes are highly expressed in infected samples and R99 samples despite they are uninfected. Cluster 1 genes are enriched in photosynthesis and light responsive process, which represent the normal physiological role of epidermal cells, while cluster 2 genes are enriched in stress response and nutrient utilization (Figure 5b), suggesting a shift from normal physiological conditions to stress responses across the pseudotime trajectory. Besides, among the cluster 2 genes, we found a series of genes that are induced during infection in N110 were highly expressed in the R99 sample, such as *Zm00001eb334730*, which is the homologue of rust resistance kinase Lr10 (*LRK10*) and RPP13‐like protein 1 (*Zm00001eb405770*). Multiple studies have shown that these highly expressed genes play a positive role and were also an important part of the signal path by which plants activate a series of defensive feedbacks after it is infected by pathogen (Liang *et al*., 2023; Prigozhin and Krasileva, 2021; Xia *et al*., 2020; Yang *et al*., 2016). The pseudotime trajectory analysis further demonstrated that the expression of specific DEGs associated with resistance in R99 was higher than in the susceptible N110 (Figure 5c). This finding underscores the superior immune response of R99 compared to N110 following pathogen infection, highlighting the importance of pre‐activated defence mechanisms in plant disease resistance. The similar pre‐activated immune states in guard and epidermal cells prompted us to examine whether there are intersected genes that are induced in R99 in both cell types. We found 23 genes that are co‐activated in both cell types (Table S8), including the AP2 transcription factor (*Zm00001eb271520*), which is known to confer resistance to biotic stress.

### Fungi induced chitinase gene *ZmCHit7* ^*R99*^ enhances resistance to *P. polysora* in maize leaves

**FIGURE 6:**
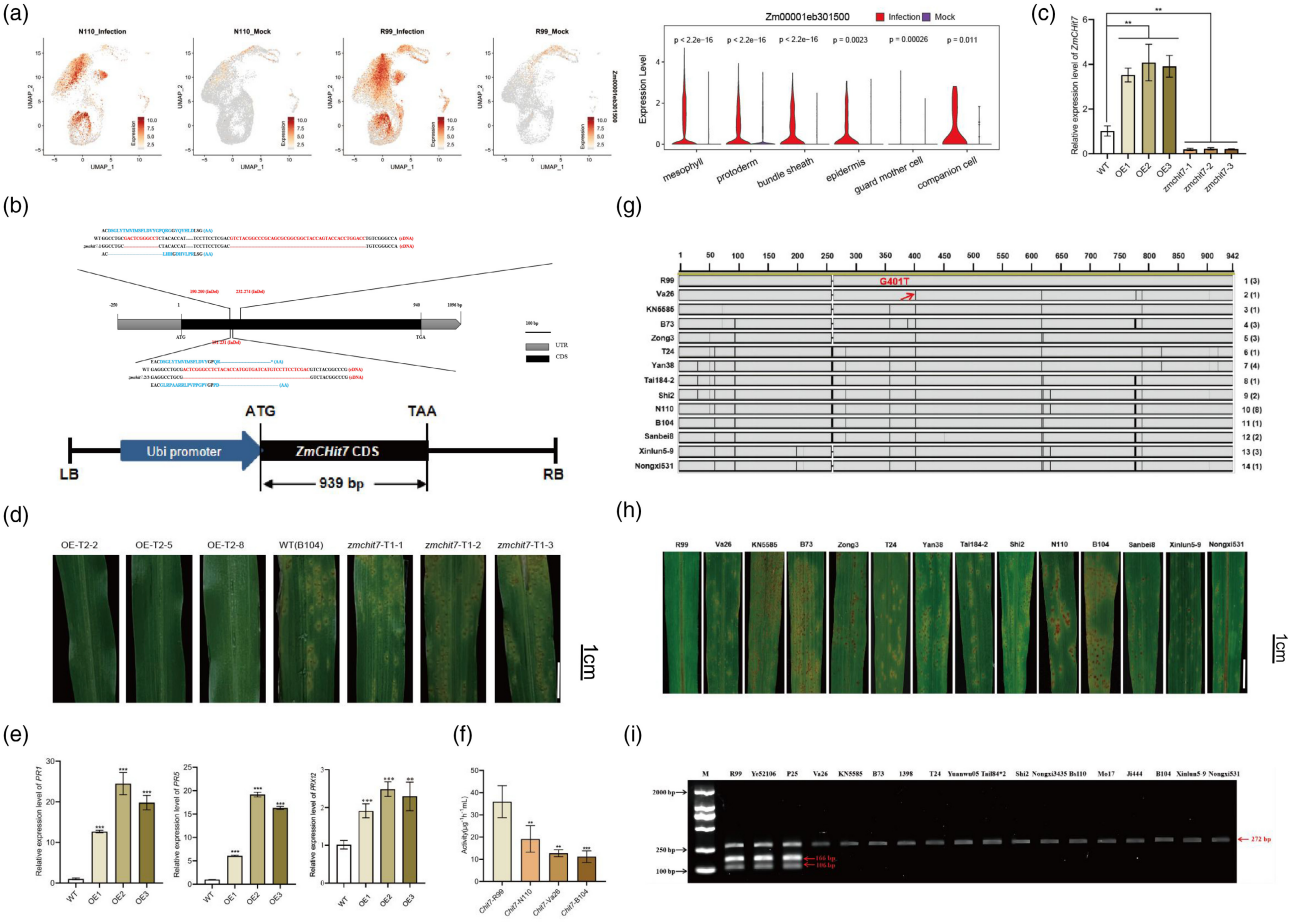
Validation of *ZmCHit7* with a stable transformation, haplotype analysis and phenotype display. (a) Feature plot of *ZmCHit7* gene expression levels under different conditions (mock, infection, R99 and N110). The violin diagram showing the expression level of *ZmCHit7* expression across cell types in infected and mock condition. (b) *ZmCHit7* coding sequence (CDS) construct used for transformation of susceptible line B104. Ubi, maize polyubiquitin gene promoter; LB, left border; RB, right border. And three *Zmchit7‐R99* knockout lines by generating indels through CRISPR/Cas9, the editing sites and amino acids change were indicated. (c) RT‐PCR showing the expression levels of *Zmchit7‐R99* overexpression and knockout lines. (d) Post‐inoculation responses of T_1_ plants to *P. polysora*, in transgene and knockout lines. Photographed at two weeks post inoculation. (e) RT‐PCR results showing the induction of *ZmPR1*, *ZmPR5* and *ZMPRX12* genes in three *Zmchit7*‐R99 overexpression lines. (f) Chitinase activity in with Zmchit7 sequences derived four representative haplotypes. (g) Alignment of 14 haplotypes (grey rectangles) identified in maize accessions through resequencing *ZmCHit7* CDS. Haplotypes 1 and 10 were from resistant line R99 and susceptible lineN110 used in genetic analysis. Vertical black lines and empty areas inside the rectangles indicate positions of SNPs and small insertions and deletions (indels), respectively, compared with haplotype 1. Numbers on the right are numbers allocated to each haplotype and numbers in parentheses indicate the number of accessions with the same haplotype, compared with haplotype 1. (h) Responses of 2‐week‐old seedlings of R99, N110 and 12 cultivars inoculated with *P. polysora*. (i) Validation of the functional CAPS marker *ZmCHit7‐EcoO109I* for *ZmCHit7* across a panel of resistant and susceptible maize accessions. Note: OE1, OE‐T2‐2; OE2, OE‐T2‐5; OE3, OE‐T2‐8; WT, B104; *zmchit7*‐1, *zmchit7*‐T1‐1; *zmchit7*‐2, *zmchit7*‐T1‐2; *zmchit7*‐3, *zmchit7*‐T1‐3.

Genes whose expression is induced by pathogen infection may play a role in plant resistance. Among the genes induced in multiple cell types in both maize varieties, we focused on a chitinase gene (*Zm00001eb301500*), which encodes a glycoside hydrolase family 18 protein. Chitin is known to trigger PTI, leading to stomatal closure and enhanced disease resistance, and is utilized in crop disease management strategies (Cheng *et al*., 2017; Jiang *et al*., 2002; Liu *et al*., 2019; Miya *et al*., 2007; Wan *et al*., 2008). Interestingly, our study revealed that both R99 and N110 highly expressed the chitinase gene *Zm00001eb301500* in multiple cell types under SCR infection (Figure 6a), suggesting that its induction is a general response of the plant's innate immunity, rather than a specific interaction between pathogen effector genes and *R* genes.

The Chitinase gene (*Zm00001eb301500*), tentatively designated as *ZmCHi7*, is 939 base pairs (bp) in length, which encodes 311 amino acids with a GH18‐hevamine‐XipI‐class‐III domain and a GH18‐ chitinase‐like superfamily (Figure S7a,b). Hevamine, a class III endochitinase, plays a vital role in defence by hydrolyzing chitin and peptidoglycan in pathogenic bacteria and fungi (Liu *et al*., 2023). Notably, numerous SNPs and InDels differentiate *ZmCHit7* ^*R99*^ from its counterpart in the susceptible N110 line (*ZmCHit7* ^*N110*^) (Figure S7c). Under fungal infection, chitin levels in R99 progressively increased, in contrast to a decrease in N110 (Figure S7d). At 24 h post‐inoculation, R99 exhibited significantly higher chitin levels than N110, corroborating R99's resistance to southern rust (Figure S7d).

To validate if *ZmCHit7* ^*R99*^ was sufficient to confer SCR resistance, we generated both three *ZmCHit7* ^*R99*^ overexpression maize lines and three knockout lines and confirmed expression changes of *ZmCHit7* ^*R99*^ in the corresponding materials (Figure 6b,c). Notably, the *ZmCHit7* ^*R99*^ overexpression conferred resistance to SCR, while the knockout lines were more sensitive to SCR (Figure 6d; Figure S7e), suggesting that *ZmCHit7* ^*R99*^ positively regulates maize resistance to SCR. Further, we found that *ZmCHit7* ^*R99*^ overexpression induced the expression of pathogenesis related genes, such as *ZmPR1*, *ZmPR5* and *ZmPRX12* (Figure 6e). Furthermore, we also performed RNA‐seq in two *ZmChit7*‐R99 overexpression lines with three replicates, respectively. We found that *ZmChit7*‐R99 overexpression activated pathways like plant hormone signalling, plant pathogen interaction and MAPK signalling, which are beneficial for fungi resistance (Figure S7f). To explore whether there are any sequence variations that potentially contribute to the differences of resistance in R99 and N110. Further analysis was conducted to elucidate sequence variations at the *ZmCHit7* locus. Examination of Chitinase activity in with *Zmchit7* sequences derived four representative haplotypes among maize inbred lines suggested that the sequence variations in *Zmchit7* substantially affect their enzyme activity (Figure 6f). The full‐length coding sequence (CDS) of *ZmCHit7* was amplified from 34 maize accessions, including R99 and N110. Accessions R99, Ye52106 and P25, which harbour the *ZmCHit7* ^*R99*^ allele, demonstrated SCR resistance, whereas the remaining 31 accessions were susceptible and exhibited considerable sequence variability compared to the resistance allele (Figure 6g–i). Sequence alignment identified 14 primary haplotypes and a pivotal single‐nucleotide polymorphism (SNP) G401T, distinguishing the resistant allele (G) from the susceptible alleles (T) (Figure 6g–i). Based on this key SNP (c. G1401T, p. R135L), a functional cleaved amplified polymorphic sequence (CAPS) marker, *ZmCHit7‐EcoO109I*, was developed to facilitate the screening of the *ZmCHit7* ^*R99*^ gene and its alleles (Figure 6g). We have surveyed in total 180 maize lines using the CAPS and found that the maize lines GG genotypes was much less infected by SCR compared to the lines with TT genotypes (*P* value <0.001, Table S9), further confirmed the power of this maker in the selection of SCR resistance line.

## Discussion

Southern corn rust (SCR), caused by the biotrophic fungal pathogen *Puccinia polysora*, poses a significant threat to corn (*Zea mays* L.) yields worldwide. The timing of disease onset relative to plant development is a critical factor influencing yield outcomes (Kleczewski and Geisler, 2022). In the event of an outbreak, the absence of both qualitative and quantitative resistance intemperate maize germplasm can lead to considerable yield losses (Brewbaker *et al*., 2011). Consequently, the identification and incorporation of resistance genes into breeding programs remain the most cost‐ effective strategy for managing this pathogen. The traditional approaches, such as genome‐wide association studies (GWAS) and bi‐parental mapping, continue to be employed to discover QTL/genes conferring SCR resistance. These methods, which integrate phenotypic screening with genotyping, enable the localization of gene loci on specific chromosomes, facilitating the identification of resistance genes. However, these techniques are time‐intensive, particularly for large populations. This study represents a paradigm shift by employing single‐cell sequencing technology to elucidate gene expression dynamics in maize leaf cells during *P. polysora* infection. We have successfully identified resistance genes to SCR, marking a novel milestone in understanding maize's resistance mechanisms at the single‐cell level. Our findings also highlight the pivotal role of *ZmCHit7*, a GH18 domain‐containing protein, in mediating SCR resistance, likely through a hormone‐mediated defence pathway.

Single‐cell transcriptomics offers an unprecedented resolution of plant disease resistance processes at the individual cell type level, independent of histological or genetic markers (Liang *et al*., 2023; Satterlee *et al*., 2020). This technique has enabled us to identify resistance genes and delve deeper into the host‐pathogen interaction's molecular mechanisms. In our study, high‐quality protoplasts were isolated from the leaves of both resistant (R99) and susceptible (N110) lines post‐infection, capturing the major cell types. A cluster‐specific marker gene library was constructed to categorize maize leaf tissues into distinct cell types, revealing that immune‐related gene ontology (GO) terms were predominantly upregulated in the epidermis, guard and mesophyll cells. In this study, we observed that host cells exhibit distinct gene expression patterns during the pre‐haustoria and post haustoria phases of *P. polysora* infection. Notably, genes involved in photosynthesis were significantly downregulated in epidermal cells post‐infection, while those associated with glucose utilization saw an increase. This shift suggests are programming of cellular processes in response to pathogen attack. The disease resistance RPP13‐like protein (*Zm00001eb405770*) has been identified as a key regulator for heat‐stress tolerance and disease resistance in plants (Rose *et al*., 2004; Yang *et al*., 2021), highlighting the intricate network of plant defence mechanisms.

The differential proteins identified across all cell clusters, including chitinase, beta‐glucosidase, cysteine‐rich receptor‐like protein kinase, calcium signalling, redox regulation and jasmonates, are directly related to disease resistance. Understanding the regulatory mechanisms controlling the expression of these genes is crucial for unravelling the molecular basis of the maize *P. polysora* interaction. Phenotypic identification, histological observation and GO enrichment analysis will further elucidate the differences in protein accumulation between the resistant line R99 and the susceptible line N110 following *P. polysora* infection, suggesting a role for these genes in the interaction.

Chitinase, a group of proteins expressed by plants under pathogenic attack, is secreted across various plant tissues and plays a pivotal role in the biodegradation of chitin. It is involved in multiple physiological and biochemical processes, including defence against fungi and bacteria (Viterbo *et al*., 2002). For instance, the chitinase gene *RmChi44* has demonstrated antifungal activity against phytopathogenic fungi (Yang *et al*., 2016). Our research identified the SCR‐resistant gene *ZmCHit7* through single‐cell sequencing combined with GO enrichment analyses. *ZmCHit7*, which contains aglycosyl hydrolase family 18 (GH18) domains, was found to be downregulated in response to photosynthesis and chloroplast organization in specific cell types post‐infection. This aligns with previous findings by Wang *et al*. (2019), who reported a decrease in photosystem subunits and chlorophyll‐binding complexes in susceptible genotypes post‐infection. Moreover, *ZmCHit7* transgenic plants exhibited fewer spores compared to wildtype lines, confirming its role in enhancing SCR resistance in maize.

Haplotype analysis using 34 common Chinese maize inbred lines revealed that the highly resistant lines contained the *ZmCHit7* gene, whereas the susceptible lines did not or only possess the *ZmCHit7* gene allele. Currently, *ZmCHit7* continues to demonstrate effective resistance in the field in China. Consequently, the functional marker *ZmCHit7‐109I*, developed in this study, holds promise for marker‐assisted selection in breeding programs and for the postulation of *ZmCHit7* in maize germplasm.

## Materials and methods

### Plant materials and *Puccinia polysora* inoculations

R99 is a homozygous inbred line of tropical germplasm introduced from abroad after excellent domestication. Nongxi 110 (N110) is a temperate resource selected at the Hebei Agricultural University Experimental Station in Baoding City, Hebei province. To investigate the effects of *P. polysora* infection on maize, inbred lines R99 and N110 underwent a phenotype examination. Plants were grown in a greenhouse under a 16 h light/8 h (25 °C) photoperiod to 3 leaf stages. Then, those plants were inoculated with a solution consisting of 0.02% Tween‐20 (v/v) and approximately 100 *P. polysora* spores per mL. *P. polysora* spores were collected from *P. polysora* infected susceptible adult N110 plants exhibiting SCR symptoms. The seedlings were immediately placed in plastic covered cages and incubated in darkness at 20 °C and 80% relative humidity (RH) for 24 h. They were then moved to a growth chamber with 16 h light/8 h darkness at 22 to 25 °C and 70% RH. SCR disease symptoms were recorded 10–14 days post inoculation (dpi) at the seedling and 20 days post‐pollination at the adult stages, using the 1–9 scale as modified by Deng *et al*. (2019).

### Observation and slicing of the microstructure of maize leaves

For the visualization of the effect of biological stress on the microstructure of the R99 and N110 seedling leaves, three‐leaf stage seedlings infected with *P. polysora* were harvested at 4 days and propidium iodide (PI) stained using the WGA‐AF488/PI cell staining method. First, the sample was equilibrated with 1 μg mL^−1^ propidium iodide, 10 μg mL^−1^ WGA‐AF 488, 0.02% Tween 20 in PBS pH 7.4 and standby. After fixing the sample with Carnot fixative, the infected leaf material was bleached in pure ethanol. The infected leaves were then transferred to a 10% KOH solution, sealed and incubated at 85 °C for 4 h, and washed 4–5 times with 1xPBS buffer (pH = 7.4); staining solution was added and vacuumed 4 times (under 250 mbar conditions) for 5 min each time, with an interval of 5 min at atmospheric pressure. After 2–3 washes with PBS, the infected leaves were sealed with anti‐fluorescence quenching sealing agent. Finally, images were analysed using an OLYMPUS BX53F fluorescence microscope (Olympus, Tokyo, Japan).

### Protoplast preparation and isolation for scRNA‐seq

The maize leaves were harvested at 12 h post‐inoculation (hpi) with or without *P. polysora* inoculation, with two biological replicates for each condition; then high‐quality protoplasts suitable were used for subsequent single‐cell transcriptome experiments. For the extraction of maize leaf protoplasts suspensions, the infected leaves were detached at 12 hpi, using modified methods to those of Luo *et al*. (2022). Briefly, the leaves were cut into 0.5‐1 mm pieces and placed in a conical flask containing 20 mL of enzymatic hydrolysate 1.5% Cellulase R10, 0.5% Hemicellulase, 0.75% Macero‐zyme R10, 0.8 M D‐mannitol 10.93 g, MES (2‐(N‐morpholino) Ethanesulfonic acid, 0.03 g CaCl_2_ and 0.02 BSA). Dark vacuum extraction for 30 min; 28 °C, 60 rpm, 2.5 h to obtain cell protoplasts. Finally, the enzymatic hydrolysis product was diluted with W5 buffer solution.

### scRNA‐seq library preparation and sequencing

Using a haemocytometer, the density of the protoplasts suspensions was calculated and set to about 1000 cells μL^−1^. Trypan blue staining was used to measure the activity of single‐cell suspensions and protoplasts with activity levels greater than 85% were prepared for further investigation (Liang *et al*., 2023). The DNBelab C Series Single‐Cell Library Prep Set (MGI) was utilized for single‐cell RNA‐seq library preparation, including droplet encapsulation, emulsion breakage, mRNA captured bead collection, reverse transcription, cDNA amplification and purification. Indexed libraries were constructed according to the manufacturer's protocol. The sequencing libraries were quantified by Qubit ssDNA Assay Kit (Thermo Fisher Scientific). The sequencing libraries were sequenced by the DNBSEQ T7. The raw data were converted to FASTQ files and underwent alignment and count quantification with DNBC4 tools software.

### Single‐cell RNA‐seq data processing

The raw gene‐count matrices were imported into Seurat v4.0.4 for downstream analysis. Firstly, genes that expressed in fewer than 3 cells and cells with a UMI lower than 500 or mitochondrial gene percent higher than 10% were filtered out. Secondly, we applied the function of “LogNormalize” to perform data normalization and the scale factor was set at 10000. Subsequently, variable genes detecting and data scaling were respectively processed using functions of “FindVariableFeatures” (“vst” method, 2000 features) and “ScaleData”. Next, the function of “RunPCA” was used for dimensionality reduction with dim set to 1:30. Then, the “FindNeighbors ()” with dims set to 1:30 and “FindClusters()” with resolution of 0.6 were used to construct the SNN graph and to cluster the cells. Finally, “RunTSNE ()” and “RunUMAP ()” were generated to visualize clusters (Becht *et al*., 2019; Maaten and Hinton, 2008). The cell type annotation of each cluster was combined by the following methods: (1) defined by known marker genes; (2) defined by significantly cluster‐enriched genes relative to the other clusters for all conditions. The cluster‐enriched Genes were computed by the “FindAllMarkers” function in Seurat (add Seurat package ref) with the following parameters: a Wilcoxon Rank Sum test and adjusted *P* < 0.05; above 1.414‐fold difference (log2FC = 0.25) between the two groups of cells; test genes that a minimum fraction was at least 0.25. The transcriptome contains more than 280 million reads in each sample and the average median number of UMIs per cell was 1059, and the average median number of genes per cell was 1126.

### Gene enrichment analysis

The function of “FindMarkers” was used to calculate DEGs between samples based on a dual threshold of |log2 FC (Fold change) | > 0.5. The screening condition for upregulation of expressed genes was *P*_adj_ <0.05 and log2FC > 0.5; and downregulation of differentially expressed genes was *P*_adj_ <0.05 and log2FC < −0.5. The intersections of DEGs between samples were visualized using the UpSetR package (Conway *et al*., 2017). DEGs in each sample were annotated using the biological process of gene ontology (GO) terms based on the reference genome. GO and KEGG (Kyoto Encyclopedia of Genes and Genomes) pathway analyses were performed using the cluster Profiler package (Yu *et al*., 2012).

### Pseudotime trajectory analysis

The expression matrix of the cell differentiation and the determination of cell fate were used to run a separate pseudotime analysis with Monocle 2 (v.2.10.0) (Trapnell *et al*., 2014). We identified key genes related to the disease and differentiation processes with a false discovery rate < 0.05 and grouped genes with similar trends in expression. We also performed GO enrichment analysis to uncover the regulatory network and functional genes.

### qRT‐PCR analysis

Total RNA was extracted using TRIzol reagent (Invitrogen, Carlsbad, CA, USA). First‐strand cDNA from total RNA was synthesized using PrimeScript™ 1st Strand cDNA Synthesis Kit (TaKaRa, Kyoto, Japan). Quantitative reverse transcription PCR (qRT‐PCR) was performed with TransStart® Top Green qPCR Super‐Mix following the manufacturer's instructions with a LightCycler 480 instrument (Roche, Colorado Springs, CO, USA). Primers used to evaluate transcript levels of the *ZmCHit7* candidate gene are listed in Table S10.

### Maize transformation

The candidate genes (e.g., *Zm00001eb301500*) coding sequence (CDS) was amplified from R99 using 2 × Phanta® Master Mix (Vazyme, Nanjing, China) following the manufacturer's recommendations and cloned into the pCAMBIA3301 binary vector driven by the maize ubiquitin promoter. The recombinant positive plasmid *pUbi: gene* was transformed into *Agrobacterium* tumefaciens strain EHA105. Immature embryos of a highly susceptible common maize B104 cultivar were transformed with the construct *pUbi: gene* using the maize transgenic technique, with assistance from Boride Biotechnology Co., Ltd., Shangxi, China. Specific markers were then used to detect the presence of the candidate genes in the transgenic plants.

### CRISPR/Cas9 mutant lines screening

For the construction of *ZmChit7* CRISPR/Cas9 mutant lines, gRNA sequences were selected in the *ZmChit7* coding sequence using the CRISPR‐P online platform (http://cbi.hzau.edu.cn/CRISPR2/). The gRNA sequence (GCACCGACTCGGTGCCACTTTTTCAAGTTGATAACGGACTAGCCTTATTTTAACTTGCTATTTCTAGCTCTAAAAC) was cloned into the CRISPR/Cas9 vector PEGOsCas9Pubi‐B‐orign‐360. And the vector and transgenic plants was provided by Beijing BomeiXingao Technology Co., Ltd. Transgenic plants (the maize inbred line B104) were screened for resistance and positive identification was performed by specific markers and sequencing.

### VIGS assays

The VIGS assays were performed in tobacco by adopting a published protocol (Li *et al*., 2021) with minor modifications. The aim of this study was to use Pr CMV‐mediated gene silencing to knock out NLR gene expression. First use of the SGN VIGS website (https://vigs.solgenomics.net/) to predict silent gene a snippet. Each Pr CMV constructs (Pr CMV‐gene for silencing genes, Pr CMV‐00 as control) and two helper vectors (pCMVF1 and pCMVZ3) were transformed into *Agrobacterium strain* GV3103 (pSoup‐p19) to co‐infect leaves of 4‐week‐old *N. benthamiana*. Then, the second leaf of maize inbred lines R99 was mechanically inoculated with *N. benthamiana* sap to induce *NLR* genes silencing. After 10 days, samples were taken for qPCR to detect the expression of *NLR* genes in Pr CMV‐gene and Pr CMV‐00 transgenic plants using specific primers. Finally, the newly grown maize leaves were inoculated with *P. polysora* and phenotypic observation and expression analysis were performed 10 days later.

We predicted the VIGS targeted sequences for the genes *Zm00001eb020600* and *Zm00001eb226690* using the SGN‐VIGS tool on the Sol Genomics Network website (solgenomics.net). Using Primer design and other tools (takarabio.com) to obtain VIGS fragment primers, Kpnl and Xbal as the cleavage sites of the joint. (1) *Zm00001eb020600* CDS was 855 bp in length. The VIGS fragment was 300 bp in length and located in CDS area 493–792 bp. Similarly, *Zm00001eb226690* CDS was 2835 bp in length. The VIGS fragment was 300 bp in length and located in CDS area 2038–2337 bp. (2) The RT‐PCR fragment of the *Zm00001eb020600* is 212 bp, which is located in the CDS region 52–263 bp. Similarly, *Zm00001eb226690* is 226 bp, located in the CDS region 2609–2834 bp. All of primers above are listed in Table S10.

### Allelic variation and functional marker development of *ZmCHit7*

Initially, 34 crop cultivars were selected to amplify the complete CDS region of gene *ZmCHit7*. The *ZmCHit7* sequences were assembled and aligned with the complete genomic sequence of *ZmCHit7* using BioEdit v.7.0.1 software. One key SNPs was specific to *ZmCHit7* and a functional CAPS marker was developed from the latter.

180 of maize inbred lines, including 34 maize accessions, R99 and N110, were used in resistance gene postulation. And evaluated for SCR reaction in field nurseries at Sanya Nanbin Farm in Henan province in the 2022/2023 and 2023/2024 maize cropping seasons. Field tests were conducted in randomized complete blocks with two replications. Follow normal field planting and management procedures. Conduct a phenotype investigation of southern rust disease after the full onset of Huangzaosi disease as a susceptible control, adopting standards of 1–9 levels.

### Chitin treatments

R99 and N110 plants were grown in a greenhouse under a 16‐h light/8‐h dark photoperiod to the two‐to three‐leaf stage were inoculated with *P. polysor* as previously described. Then we selected the samples before inoculation (0 h) and 12, 24, 48 and 96 h post inoculation (hpi) with *P. polysor* as the test materials and used the enzyme‐linked immunosorbent assay (ELISA) to determine them, using the plant chitin enzyme‐linked immunosorbent assay (ELISA) methods (Jining, Shanghai, China), respectively. The analysis was performed using three biological replicates.

## Conflict of interest

The authors declare that they have no conflicts of interest.

## Author contributions

HD and XY conceived the project. XY, QL and ML designed the experiments. XY, QY, XZ and MK performed the experiments. XY, KW and ST analysed the results and wrote the manuscript. JL and SD assisted Plotting, Investigation. XY, SD, ML and HD provided scientific suggestions and revised the manuscript. All authors reviewed and approved the final manuscript. Data availability statement: The sequence data have been deposited in the National Center for Biotechnology Information Short Reads Archive (PRJNA1085690). Plant materials are available upon request.

## Supporting information


**Figure S1.** (a) Representative protoplast quality for generating single‐cell data (b) Scatter plot showing the correlation between biological replicates at pseudo bulk tissue level. (c) Heatmap showing the correlation between biological replicates at cell type levels.
**Figure S2.** The proportion of each sample grouping diagram and cell type.
**Figure S3.** The expression patterns of representative top 10 cell type‐specific marker genes in 6 major cell types. One feature plot representing the marker for each cell type.
**Figure S4.** Identification of the intersection of up or down‐regulated DGEs by N110_infection_vs_mock and R99_infection_vs_mock using Venn diagrams.
**Figure S5.** (a) UpSet plot showing the NLR genes that exhibited significant changes in different comparison groups (b–d) Heatmap showing NLR genes that exhibited significant changes in different comparison groups.
**Figure S6.** Heatmap shows average expression of 204 *NLR* genes in different cell types under different conditions. The colour bar indicates the relative expression level.
**Figure S7.** (a, b) Deduced amino acid sequence of *ZmCHit7*. GH18_hevamine_XipI_class_III domain indicated in red script. Domains were predicted based on NCBI search programs (https://www.ncbi.nlm.nih.gov/Structure/cdd/wrpsb.cgi). (c) Amino acid sequence comparison of 14 haplotypes (grey rectangles) identified in maize accessions. (d) Determination of the content of chitin in middle leaves of maize before and after inoculation with R99 and N110 during the trefoil stage. (e) Number of rust spores in *ZmCHit7* overexpression and Crispr/Cas9 knockout lines. (f) KEGG enrichment analysis of genes significantly up‐regulated in *ZmCHit7‐R99* overexpression lines.

**Table S1.** Quality control Summary of each sample.
**Table S2.** Statistical table of cell counts in each subpopulation of each sample.
**Table S3.** List of cell‐type marker genes used in this study.
**Table S4.** Genes indicate the identified top10 marker genes for each cluster.
**Table S5.** Genes differentially expressed between R99 and N110 in each cell types.
**Table S6.** Atotal of 204 *NBS‐LRR* genes expressions were detected in our expression profile.
**Table S7.** *NLR* Genes indicate the identified top10 marker genes for each cluster.
**Table S8.** Genes that are co‐activated in guard and epidermal cells in R99 compared to N110.
**Table S9.** The G401T genotypes and SCR infection severity across 180 maize inbred lines.
**Table S10.** Sequences of PCR primers used in the study.

## Data Availability

The data that supports the findings of this study are available in the supplementary material of this article.
