# Supplementary material for: Single‐cell transcriptomic profiling of maize cell heterogeneity and systemic immune responses against Puccinia polysora Underw
Source: Plant Biotechnol J. 2024 Nov 29;23(2):549–63. doi: 10.1111/pbi.14519 (PMC11772323; doi:10.1111/pbi.14519)
Supplement: Supplementary file 1 — Figure S1. (a) Representative protoplast quality for generating single‐cell data (b) Scatter plot showing the correlation between biological replicates at pseudo bulk tissue level. (c) Heatmap showing the correlation between biological replicates at cell type levels. Figure S2. The proportion of each sample grouping diagram and cell type. Figure S3. The expression patterns of representative top 10 cell type‐specific marker genes in 6 major cell types. One feature plot representing the marker for each cell type. Figure S4. Identification of the intersection of up or down‐regulated DGEs by N110_infection_vs_mock and R99_infection_vs_mock using Venn diagrams. Figure S5. (a) UpSet plot showing the NLR genes that exhibited significant changes in different comparison groups (b–d) Heatmap showing NLR genes that exhibited significant changes in different comparison groups. Figure S6. Heatmap shows average expression of 204 NLR genes in different cell types under different conditions. The colour bar indicates the relative expression level. Figure S7. (a, b) Deduced amino acid sequence of ZmCHit7. GH18_hevamine_XipI_class_III domain indicated in red script. Domains were predicted based on NCBI search programs (https://www.ncbi.nlm.nih.gov/Structure/cdd/wrpsb.cgi). (c) Amino acid sequence comparison of 14 haplotypes (grey rectangles) identified in maize accessions. (d) Determination of the content of chitin in middle leaves of maize before and after inoculation with R99 and N110 during the trefoil stage. (e) Number of rust spores in ZmCHit7 overexpression and Crispr/Cas9 knockout lines. (f) KEGG enrichment analysis of genes significantly up‐regulated in ZmCHit7‐R99 overexpression lines. [file PBI-23-549-s002.docx]

Figure S1


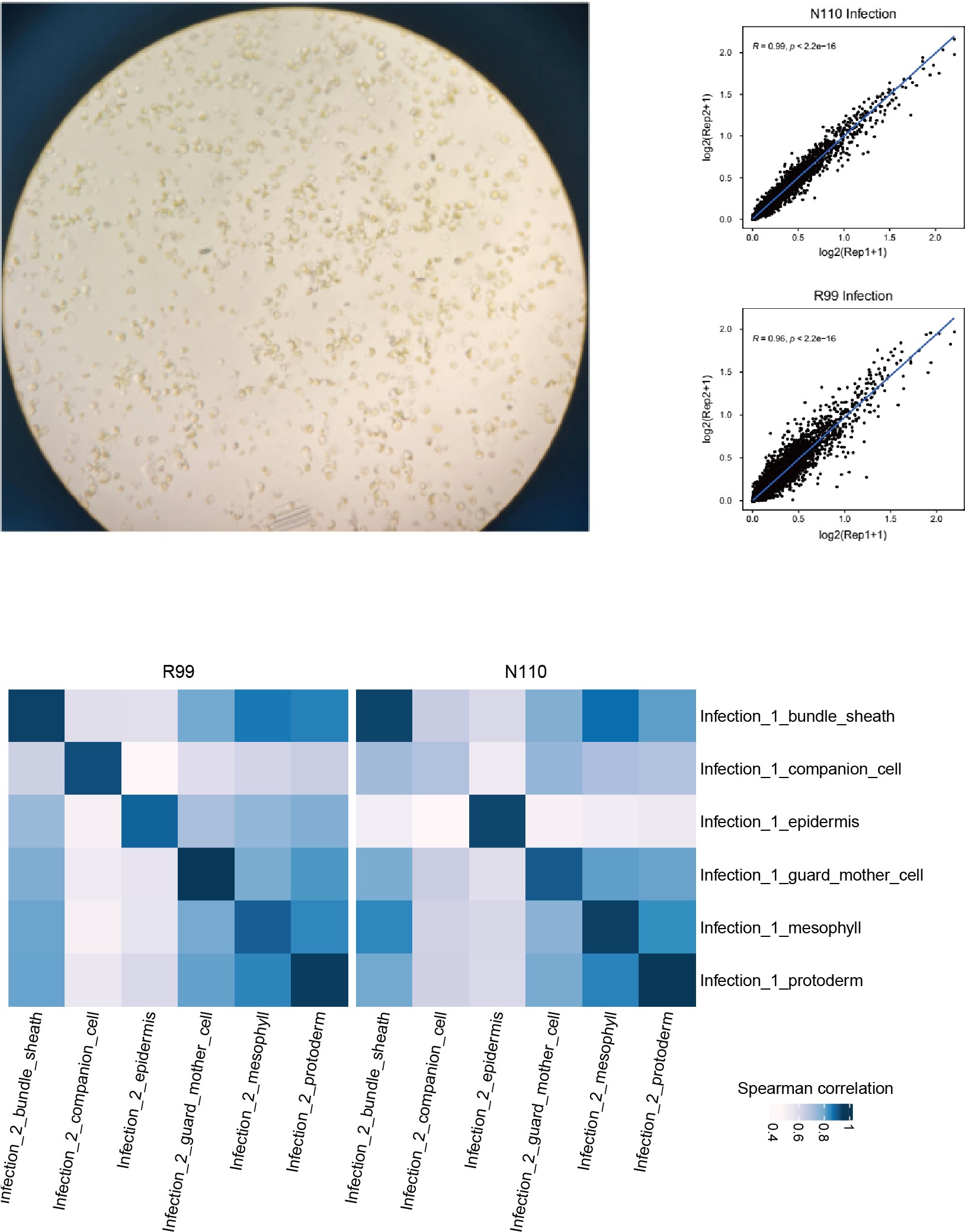
(a) (b)

(c)

Figure S2


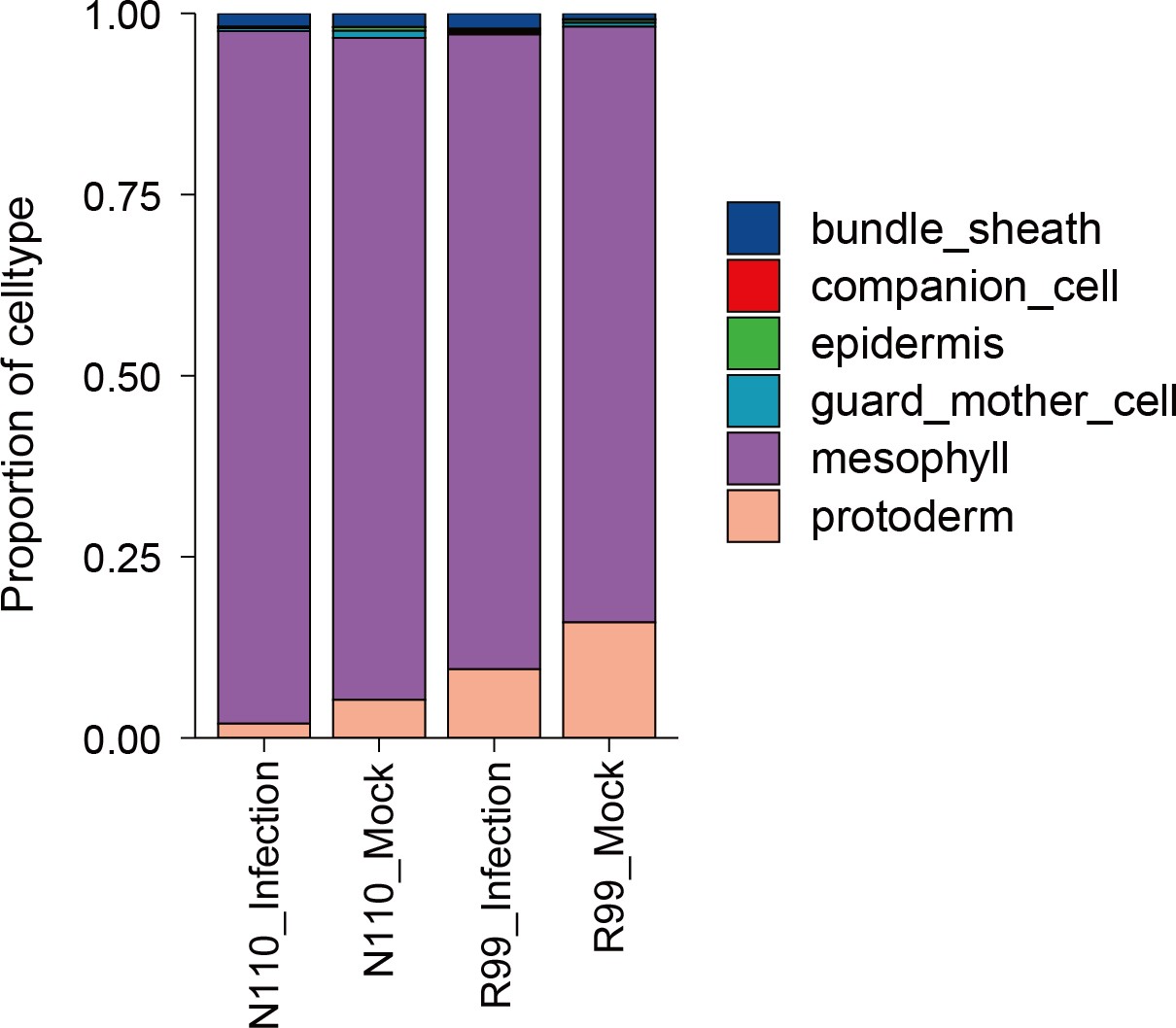


Figure S3


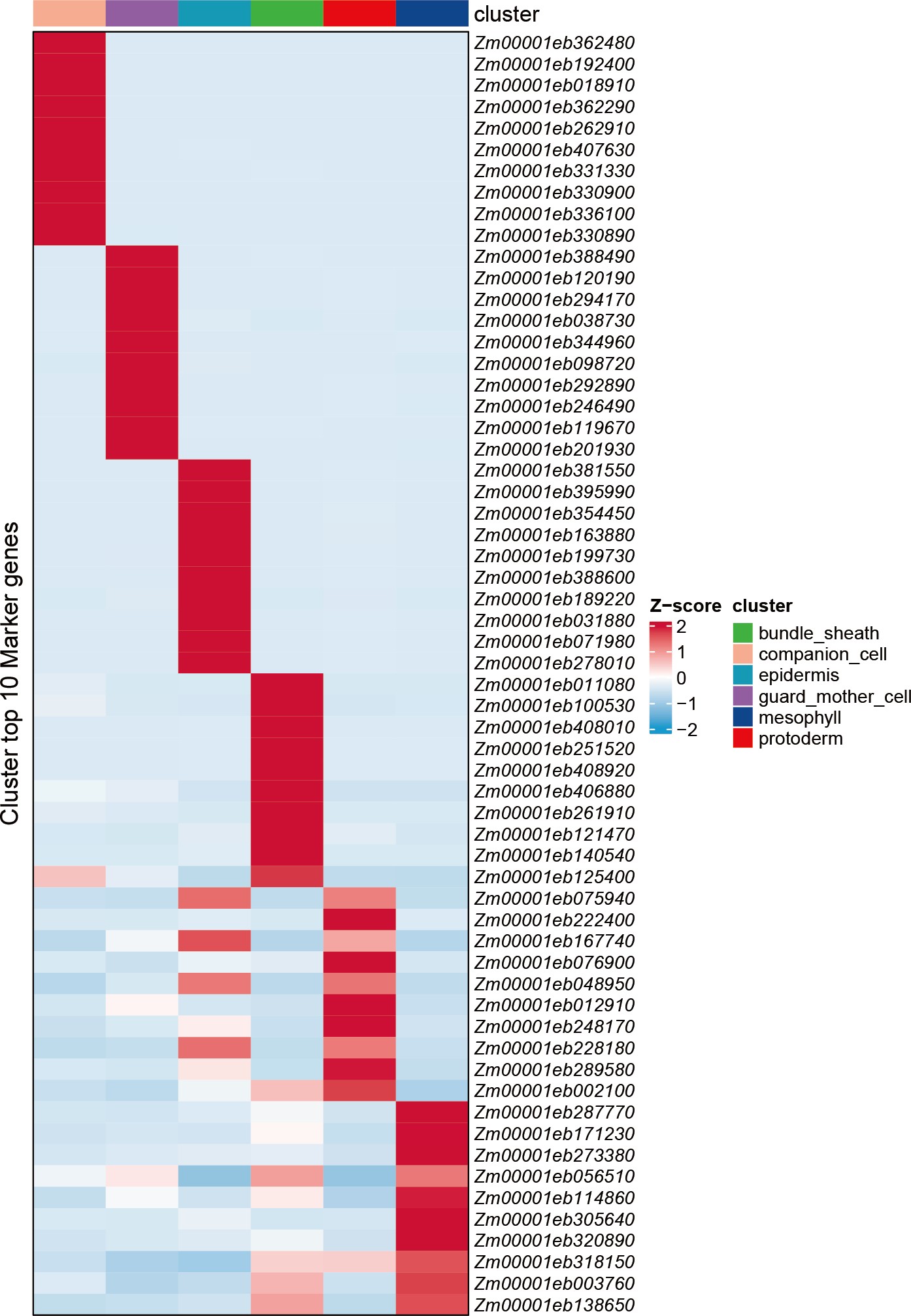


Figure S4

(a)

(b)

N110_infection_vs_mock





Up

Down

R99_infection_vs_mock

Up

Down

Figure S5

(a)


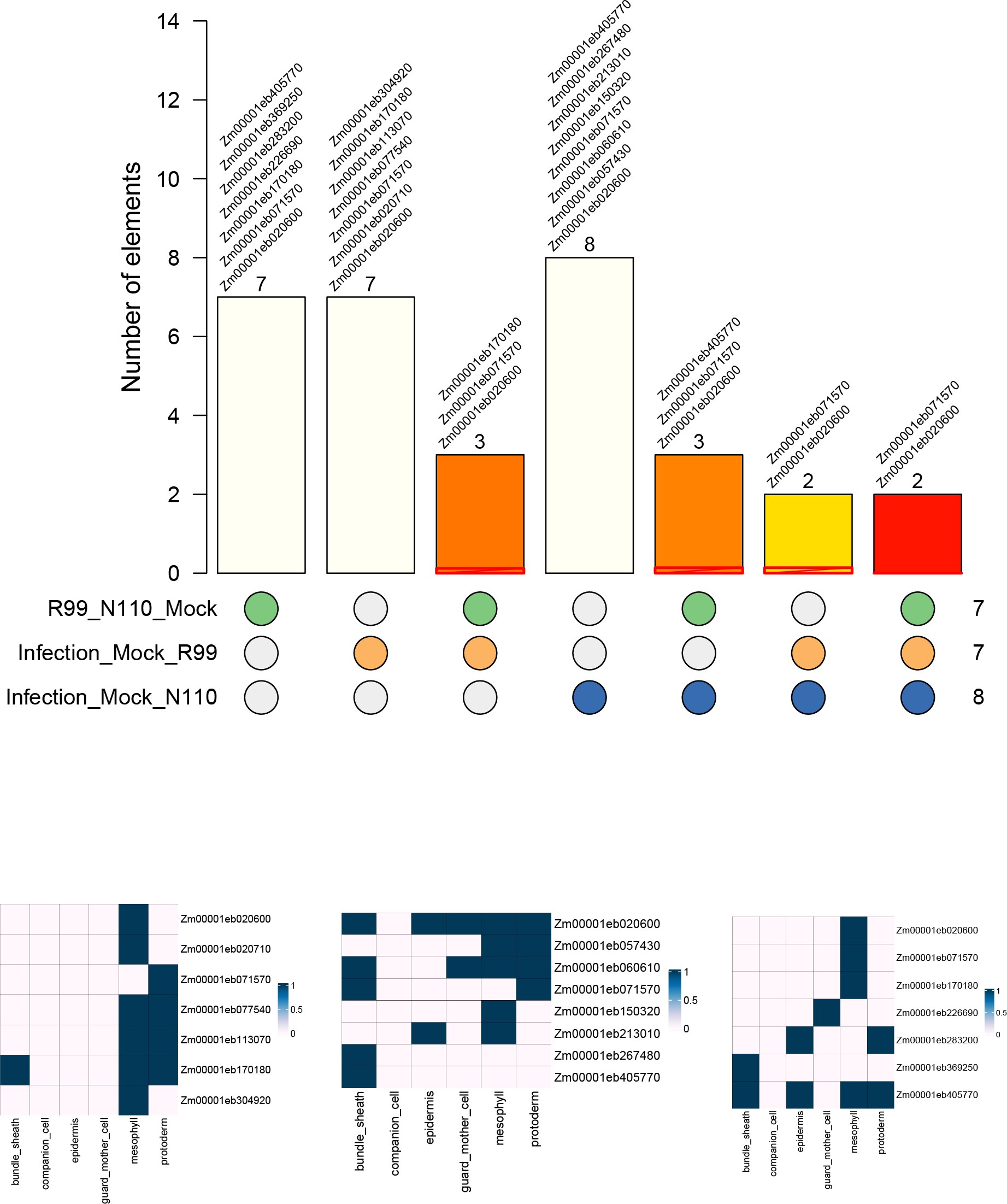
(b) (c) (d)

R99Infection_vs_Mock UP (NLR) N110Infection_vs_Mock UP (NLR) R99mock v.s. N110mock UP (NLR)

Figure S6

# line compare

Zm00001eb006270 Zm00001eb410900 Zm00001eb401510 Zm00001eb134970 Zm00001eb071570 Zm00001eb226710 Zm00001eb298830 Zm00001eb100940 Zm00001eb134820 Zm00001eb405860 Zm00001eb322860 Zm00001eb401390 Zm00001eb213010 Zm00001eb020600 Zm00001eb057430 Zm00001eb221980 Zm00001eb121910 Zm00001eb361880 Zm00001eb119560 Zm00001eb163580 Zm00001eb239020 Zm00001eb298800 Zm00001eb060610 Zm00001eb060620 Zm00001eb349360 Zm00001eb417460 Zm00001eb283200 Zm00001eb321430 Zm00001eb237210 Zm00001eb042890 Zm00001eb205560 Zm00001eb030390 Zm00001eb185570 Zm00001eb386390 Zm00001eb261660 Zm00001eb288390 Zm00001eb010990 Zm00001eb260980 Zm00001eb078010 Zm00001eb337150 Zm00001eb150750 Zm00001eb116510 Zm00001eb116200 Zm00001eb298840 Zm00001eb259320 Zm00001eb050890 Zm00001eb165170 Zm00001eb376840 Zm00001eb020710 Zm00001eb245940 Zm00001eb327200 Zm00001eb271410 Zm00001eb405370 Zm00001eb324700 Zm00001eb077540 Zm00001eb044020 Zm00001eb107150 Zm00001eb406540 Zm00001eb419360 Zm00001eb170180 Zm00001eb416830 Zm00001eb304920 Zm00001eb416940 Zm00001eb361660 Zm00001eb150780 Zm00001eb196580 Zm00001eb197290 Zm00001eb290880 Zm00001eb113070 Zm00001eb318600 Zm00001eb200120 Zm00001eb163590 Zm00001eb037410 Zm00001eb307130 Zm00001eb226700 Zm00001eb253770 Zm00001eb187220 Zm00001eb418840 Zm00001eb034880 Zm00001eb050900 Zm00001eb116330 Zm00001eb078100 Zm00001eb150770 Zm00001eb419320 Zm00001eb297430 Zm00001eb226690

**expression line**

Zm00001eb310010


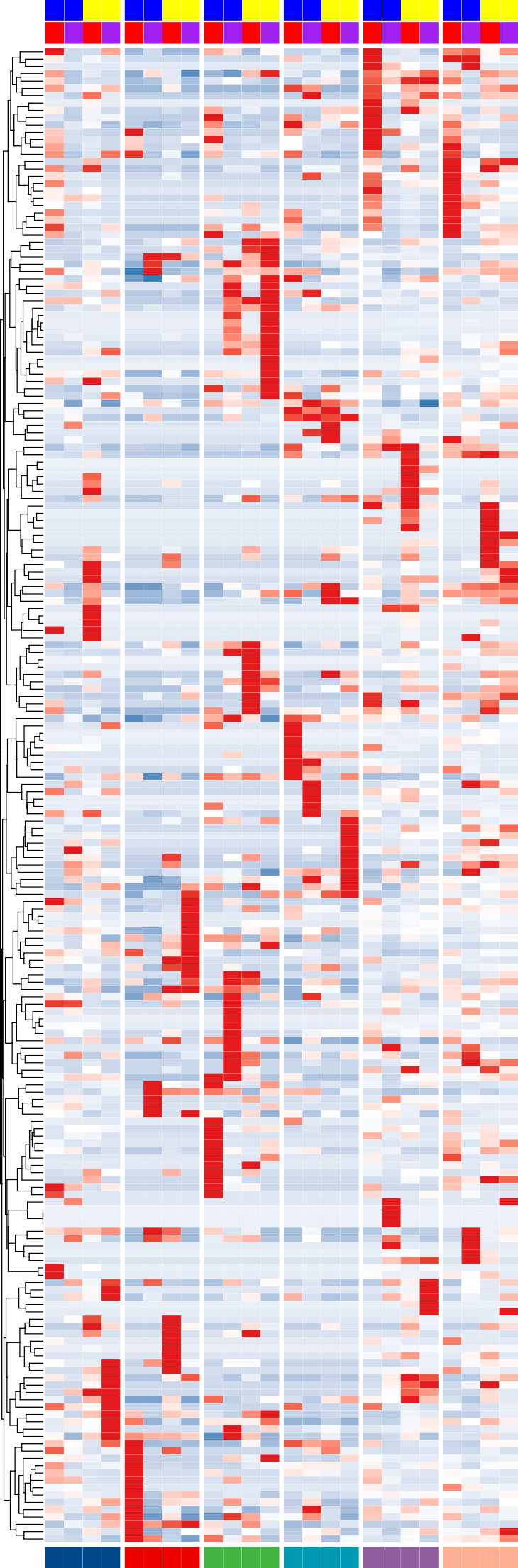

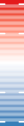

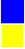
Zm00001eb199550 2

Zm00001eb100820 Zm00001eb114120 Zm00001eb145730 Zm00001eb390540 Zm00001eb321440 Zm00001eb414490 Zm00001eb224270 Zm00001eb378630 Zm00001eb159500 Zm00001eb349640 Zm00001eb165200 Zm00001eb235810 Zm00001eb261200 Zm00001eb118040 Zm00001eb115030 Zm00001eb136790 Zm00001eb267480 Zm00001eb015450 Zm00001eb200710 Zm00001eb226880 Zm00001eb256740 Zm00001eb405940 Zm00001eb184050 Zm00001eb291370 Zm00001eb163310 Zm00001eb402660 Zm00001eb349330 Zm00001eb371900 Zm00001eb024240 Zm00001eb112540 Zm00001eb223090 Zm00001eb298890 Zm00001eb424850 Zm00001eb115300 Zm00001eb369250 Zm00001eb298300 Zm00001eb161110 Zm00001eb205780 Zm00001eb126430 Zm00001eb202940 Zm00001eb046370 Zm00001eb349530 Zm00001eb283750 Zm00001eb405930 Zm00001eb401620 Zm00001eb261230 Zm00001eb405700 Zm00001eb150320 Zm00001eb091840 Zm00001eb410120 Zm00001eb137530 Zm00001eb222040 Zm00001eb151150 Zm00001eb304830 Zm00001eb137200 Zm00001eb290800 Zm00001eb091500 Zm00001eb200740 Zm00001eb118300 Zm00001eb131200 Zm00001eb006860 Zm00001eb123520 Zm00001eb322130 Zm00001eb184670 Zm00001eb138420 Zm00001eb304860 Zm00001eb137570 Zm00001eb008460 Zm00001eb091490 Zm00001eb293220 Zm00001eb283380 Zm00001eb339320 Zm00001eb353740 Zm00001eb202350 Zm00001eb200760 Zm00001eb405900 Zm00001eb261570 Zm00001eb416910 Zm00001eb383780 Zm00001eb165480 Zm00001eb405290 Zm00001eb195760 Zm00001eb405270 Zm00001eb184720 Zm00001eb199890 Zm00001eb169030 Zm00001eb174770 Zm00001eb324560 Zm00001eb207870 Zm00001eb006610 Zm00001eb108350 Zm00001eb200700 Zm00001eb227070 Zm00001eb135110 Zm00001eb361650 Zm00001eb177070 Zm00001eb228790 Zm00001eb363970 Zm00001eb405770 Zm00001eb033040 Zm00001eb354200 Zm00001eb064600 Zm00001eb035230 Zm00001eb272270 Zm00001eb200750 Zm00001eb350650 Zm00001eb094050 Zm00001eb117720 Zm00001eb113900 Zm00001eb283180 Zm00001eb247040 Zm00001eb184850 Zm00001eb273720 Zm00001eb027820 Zm00001eb226720 Zm00001eb350920

1

0

−1

−2

# cell_type

N110 R99

## compare


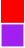
Infection Mock

## cell_type


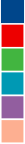
bundle_sheath companion_cell epidermis guard_mother_cell mesophyll protoderm

Figure S7

(a)

(b)

(c)

(f)


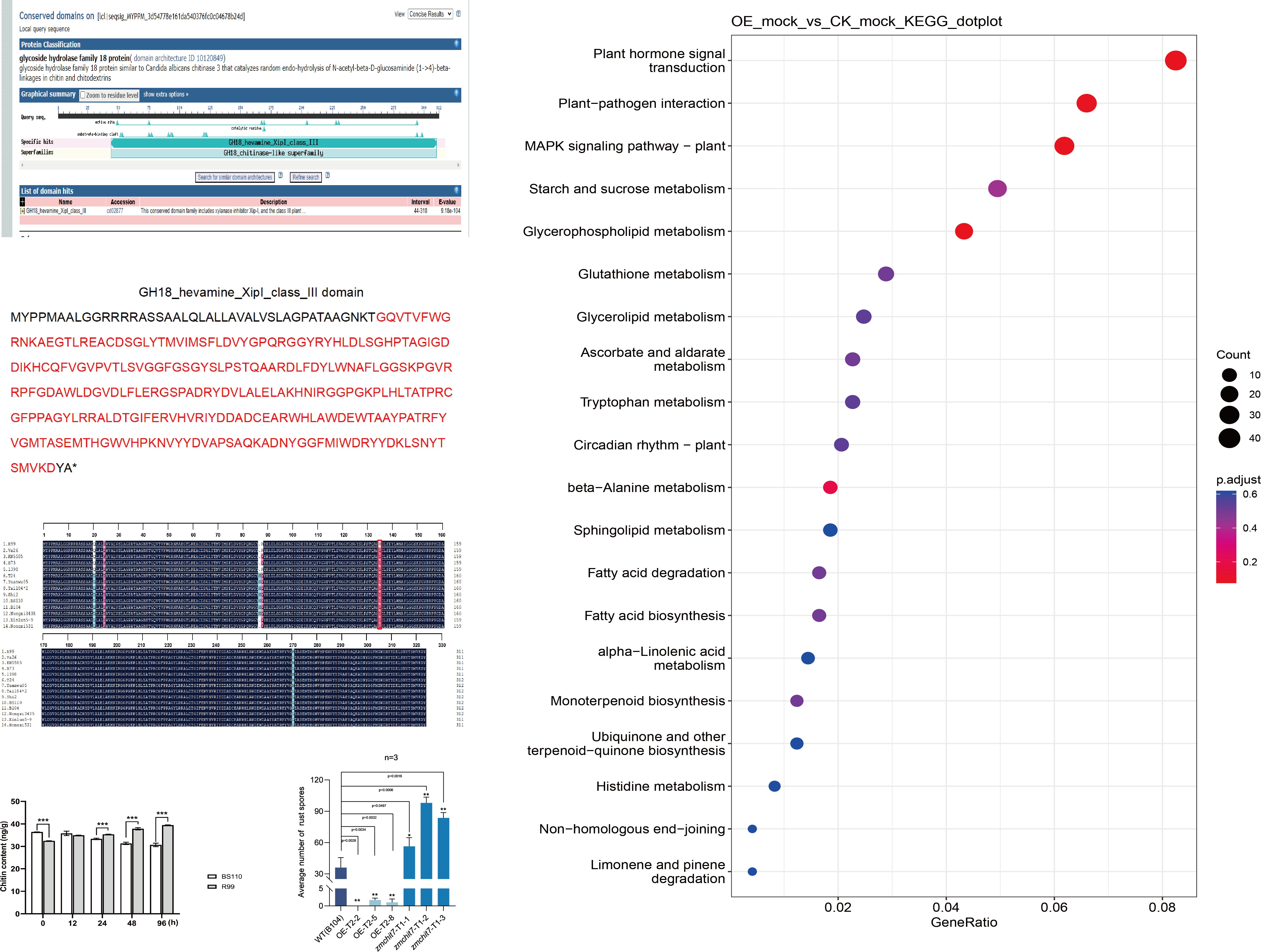


(d) (e)
